# Supplementary figures and images for: High-throughput high content quantification of HIV-1 viral infectious output
Source: PLoS One. 2026 Mar 26;21(3):e0328121. doi: 10.1371/journal.pone.0328121 (PMC13020823; doi:10.1371/journal.pone.0328121)

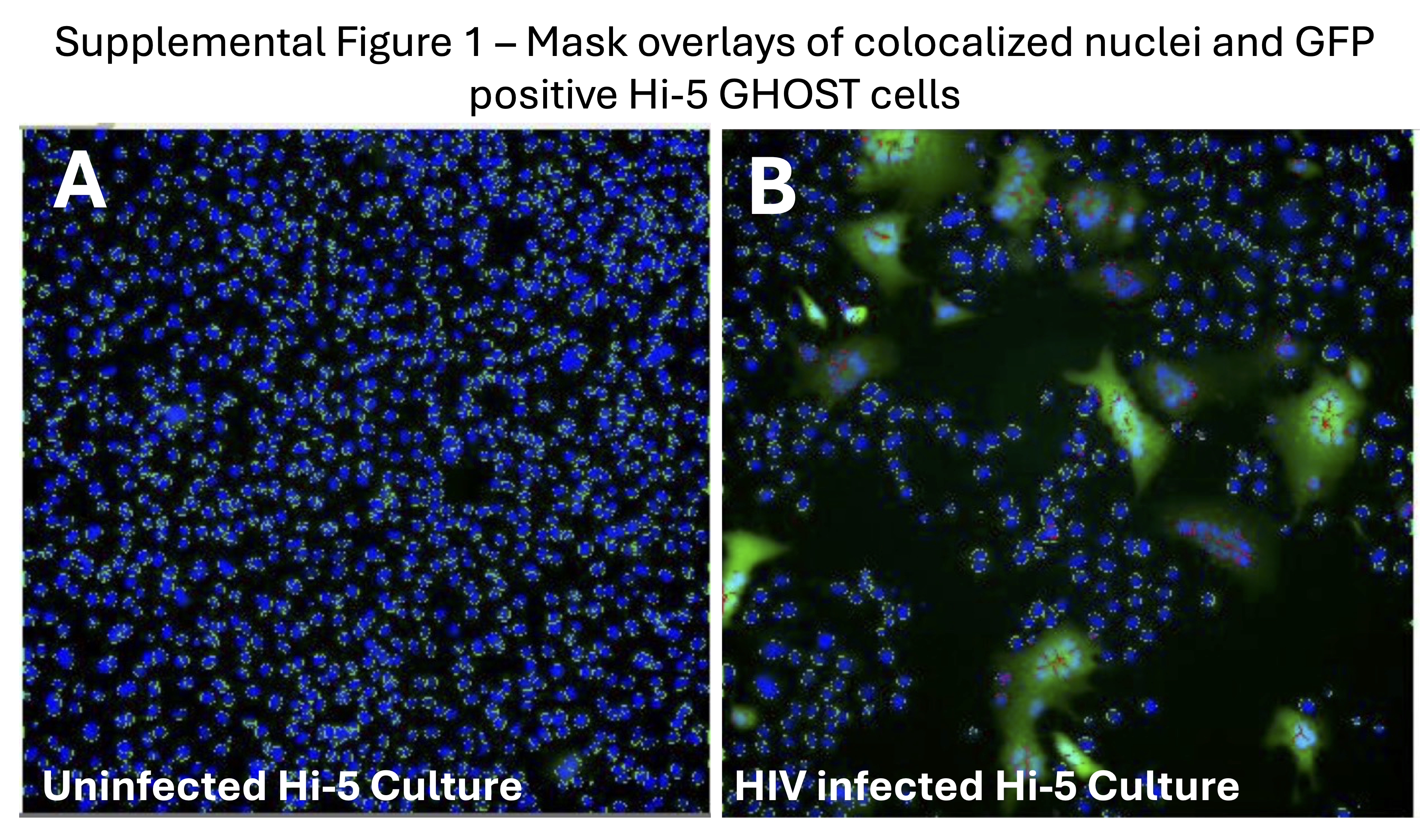

Supplement: S1 Fig — After background fluorescence subtraction, nuclei were segmented and enumerated across all fields. Colocalization analysis was then performed to identify nuclei residing within GFP-positive regions, which were designated as infected cells. Representative images illustrate the masking strategy used to define nuclei and GFP⁺ regions in uninfected Hi-5 cultures (A) and infected Hi-5 cultures (B). (TIFF) [file pone.0328121.s001.tiff]

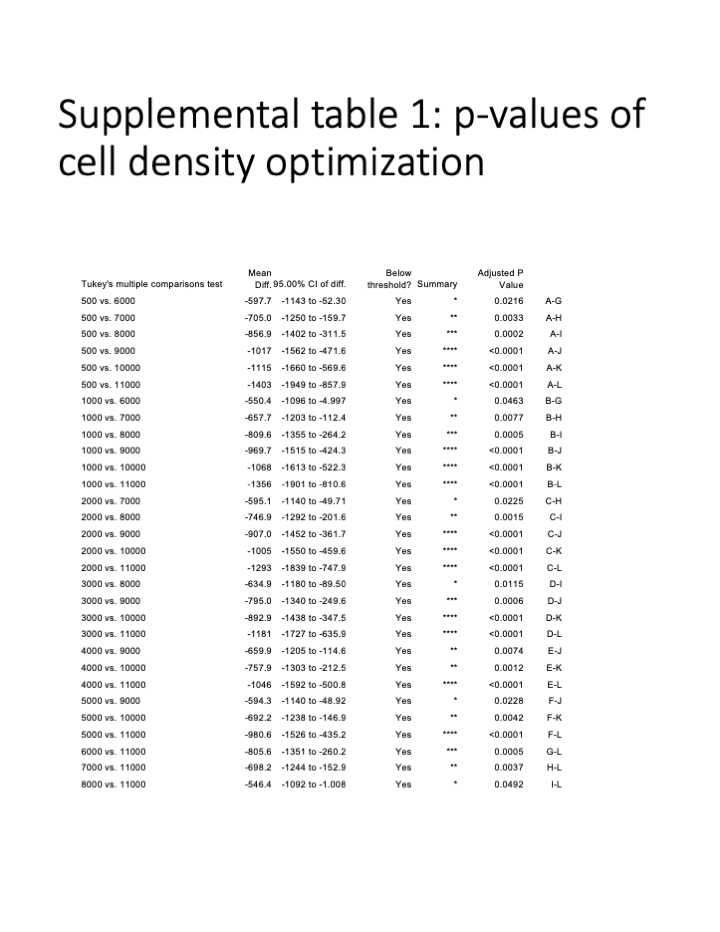

Supplement: S1 Table — Cell densities between 2,000 and 7,000 did not statistically differ from one another with significant interaction of cell density and infectious unit production. Multiple comparisons *p-values <0.05 reported for all other cell density comparisons. (TIFF) [file pone.0328121.s003.tiff]

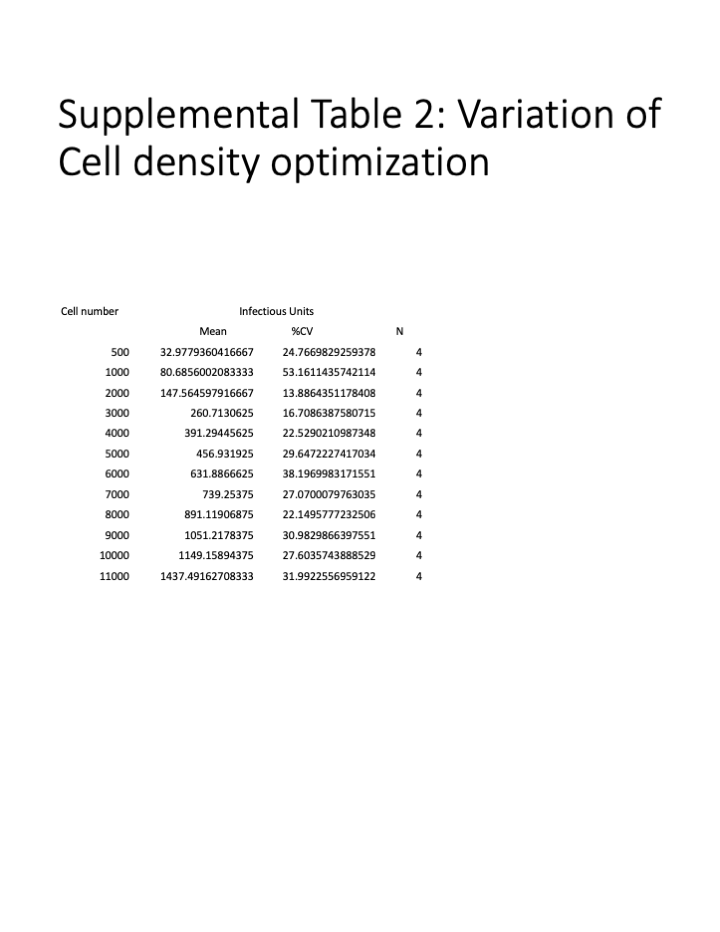

Supplement: S2 Table — Percent coefficients of variation (%CV) of the amount of IU/μL in a single sample increases between experiments with a higher cell density, with the smallest % coefficient of variation between 2,000 and 5,000 cells per well. (TIFF) [file pone.0328121.s004.tiff]

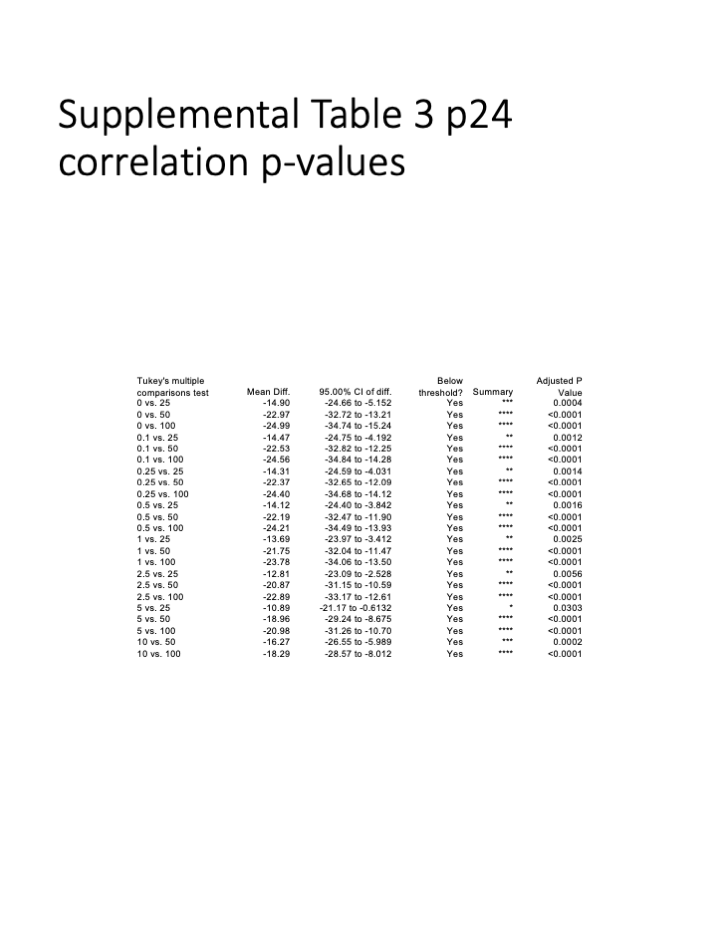

Supplement: S3 Table — This table lists all of the p-values pertaining to the comparison of infectious units to p24•Gag concentration depicted in Fig 4A-B. (TIFF) [file pone.0328121.s005.tiff]
