# Supplementary material for: High-throughput high content quantification of HIV-1 viral infectious output
Source: PLoS One. 2026 Mar 26;21(3):e0328121. doi: 10.1371/journal.pone.0328121 (PMC13020823; doi:10.1371/journal.pone.0328121)
Supplement: S1 File — (PDF) [file pone.0328121.s013.pdf]

DREXEL UNIVERSITY
Office of Research & Innovation

Office of Research & Innovation
Human Research Protection Program
Drexel University
Bellet Building
1505 Race Street, 7th Floor
Philadelphia, PA 19102

Institutional Review Board
Phone: (267) 359-2471
Fax: (215) 762-6258
E-mail: HRPP@drexel.edu

Not Human Research Determination

Date: August 5, 2022

**Protocol Number:**	2208009386
**Principal Investigator:**	Peter Gaskill, PhD
**Review Date:**	August 5, 2022
**Committee:**	IRB 1
**Sponsor:**	Pharmacology and Physiology (6326)
**Project Title:**	Benzodiazepine mediated mechanisms of transcriptional semi-quiescence in discrete myeloid populations

The proposed activity is not human subjects research as defined by DHHS or FDA regulations. Consequently, **Drexel IRB review and approval are not applicable**. You are welcome to pursue the activity, obtaining any applicable administrative or departmental (non-IRB) approvals.

This determination applies only to the activities described in this IRB submission and does not apply should any changes be made. Changes could affect this determination. Please contact the IRB for guidance.

DHHS Definitions:
Research – a systematic investigation, including research development, testing, and evaluation, designed to develop or contribute to generalizable knowledge.

Human subject – a living individual about whom an investigator (whether professional or student) conducting research:

1. Obtains information or biospecimens through intervention or interaction with the individual, and uses, studies, or analyzes the information or biospecimens; or
2. Obtains, uses, studies, analyzes, or generates identifiable private information or identifiable biospecimens.

FDA Definitions:
Research – any experiment that involves a test article and one or more human subjects, and that either: a) must meet the requirements for prior submission to the Food and Drug Administration; or b) the results of which are intended to be later submitted to, or held for inspection by, the FDA as part of an application for research or marketing permit.

Human subject – an individual who is or becomes a participant in research, either as a recipient of the test article or as a control. A subject may be either a healthy individual or a patient.

Please contact the IRB at (267) 359-2471 or HRPP@drexel.edu if you have any questions.

IRB Oversight Not Required
